# An efficient low cost means of biophysical gene transfection in primary cells

**DOI:** 10.1038/s41598-024-62996-y

**Published:** 2024-06-08

**Authors:** Shudi Huang, Tyler R. Henderson, Chesarahmia Dojo Soeandy, Anastasiya Lezhanska, Jeffrey T. Henderson

**Affiliations:** 1 Department of Pharmaceutical Sciences, University of Toronto, 144 College St. Rm 962, Toronto, ON M5S 3M2 Canada; 2 Department of Medical Genetics, Lunenfeld-Tanenbaum Research Institute, Mount Sinai Hospital, Toronto, ON M5G 1X5 Canada; 3 Tumour Immunotherapy Program Cell Manufacturing Team, Princess Margaret Cancer Centre, University Health Network, 610 University Avenue, Rm 8-207, Toronto, ON M5G 2M9 Canada; 4 Department of Medicine, Queen’s University, 94 Stuart Street, Kingston, ON K7L 3N6 Canada

**Keywords:** Gene delivery, Transfection, Biological techniques, Stem cells, Biotechnology, Gene delivery, Transfection

## Abstract

Efficient, facile gene modification of cells has become an indispensable part of modern molecular biology. For the majority of cell lines and several primary populations, such modifications can be readily performed through a variety of methods. However, many primary cell lines such as stem cells frequently suffer from poor transfection efficiency. Though several physical approaches have been introduced to circumvent these issues, they often require expensive/specialized equipment and/or consumables, utilize substantial cell numbers and often still suffer from poor efficiency. Viral methods are capable of transducing difficult cellular populations, however such methods can be time consuming for large arrays of gene targets, present biohazard concerns, and result in expression of viral proteins; issues of concern for certain experimental approaches. We report here a widely applicable, low-cost (< $100 CAD) method of electroporation, applicable to small (1–10 μl) cell volumes and composed of equipment readily available to the average investigator. Using this system we observe a sixfold increase in transfection efficiency in embryonic stem cell lines compared to commercial devices. Due to efficiency gains and reductions in volume and applied voltage, this process improves the survival of sensitive stem cell populations while reducing reagent requirements for protocols such as Cas9/gRNAs transfections.

## Introduction

Facile genetic transfection of target cell populations is an indispensable aspect of modern molecular biology, involving the introduction of DNA/RNA substituents or other cell impermeable reagents (biologic drugs, peptides, etc.) to alter signaling responses of target cells^1,2^. Often, this results in a transient or stable modification of cellular responsivity depending on whether the modification is integrated into the genome, or remains extra-genomic^1^. Particularly with the advent of CRISPR (clustered regularly interspace short palindromic repeats) and genome base-editing technologies, efficient means of performing a wide array of gene modifications have become readily available^3,4^. However the success of these and many other forms of cell modification depend upon efficient intracellular introduction of the modifying agent; the difficulty of which of varies with cell type^3^. Thus while a number of straightforward chemical and biophysical methods exist for the transfection of (particularly transformed) cell lines, such methods are limited for difficult to transfect primary cell lines such as embryonic stem (ES) cells^1,5–10^. In addition, cell types such as patient-derived primary cells from resections, biopsies, etc. are often difficult to obtain or isolate in sufficient quantity, further complicating transfection studies^11–14^. Of methods which do exist for difficult to transfect cell lines, many are often inefficient, biohazardous, and/or require use of costly specialized equipment and consumables^1,2,15^. Such considerations become significant at scale, as modifications become more complex (multiple RNA/protein targets), and in clinical settings. Though viral transduction methods (lentivirus, adenovirus, adeno-associated virus, etc.) exist to transduce difficult to transfect cell types, these too possess certain drawbacks. Despite known advantages including transduction efficiency toward non-dividing cells^15–18^, and their ability to be utilized in vivo*, *ex vivo and in vitro^1,16–19^; viral transducers require additional processing and purification in order to produce infective particles, adding time and cost particularly for operations involving a large number of target genes^15,20–22^. With respect to biosafety concerns, these include the induction of antiviral and immunologic cellular responses, potential for generation of replication-competent viruses, vector mobilization in some instances and the possibility of integration-associated oncogenesis^15,22–24^. Additionally adeno-associated virus vectors exhibit a typical insert size limit < 5 kb, limiting modifications in projects requiring multiple RNA or large DNA segments^1,18,25^.

By contrast, chemical (modified lipids, polyethylenimine, calcium phosphate) and physical (microinjection, biolistics, ultrasound, electroporation, nucleofection, cell squeezing and laser-poration) techniques are technically straightforward, more adaptable, less time-consuming and do not pose biohazard risks to laboratory personnel^1,18,26–30^. For example lipofection and polyethylenimine mediated transfection are quick, easy procedures based on the condensation of nucleic acids with cationic lipids or organic polyamine polymers respectively to facilitate fusion-based phospholipid entry into target cells^1,15^. These techniques however demonstrate variable efficiencies depending on cell type. For example typically only 1–5% of primary neurons are transfected using lipofection, despite up to 85% efficiency in many transformed cell lines^2,15^. Additionally these agents demonstrate significant toxicity toward sensitive cell types such as embryonic stem cells and neurons^1,7^. Alternatively with respect to physical methods, some difficulties include the relatively high input and per unit costs for modifications due to equipment requirements (microinjection, biolistics, ultrasound, electroporation, nucleoporation, laser-poration), consumables (nucleofection, cell squeezing, electroporation, microinjection), temporal efficiency (microinjection, laser-poration), and/or sustained throughput (microfluidic channel /cell squeezing)^26–30^.

In theory, electroporation (EP) is a fast simple method involving exposure of cells to brief electric pulses inducing pore formation through the plasma membrane in order to allow plasmids and other ectopic molecules to enter the cell cytoplasm^1,2,15^. However the electrode gap distance (4–10 mm) and associated voltages (200–800 V) typically utilized for mammalian cells require significant electrical capacity with substantial electrolytic effects (acidification, alkalinization) at anode and cathode interfaces respectively with such effects potentially causing significant damage to sensitive cell types^31^. Additionally larger chamber sizes (and thus electrode gap distance) can reduce homogeneity of the applied electric field, ultimately decreasing cell viability and transfection efficiency^32,33^. Minimum electroporation volumes required in these systems (200–1000 μl) can impart additional significant reagent costs (outside of equipment/consumable costs) for gene-editing experiments utilizing ribonucleoprotein complexes containing defined RNA guides plus Cas9, single-stranded DNA deaminase (base-editing) or equivalents (CRISPR), which must be maintained at critical concentrations for maximum efficiency. Given the current application range and relative efficiency of the above gene modification approaches, considerable interest has arisen in more cost effective, efficient means of performing such biophysical gene transfection.

We describe herein a novel, efficient, low-cost means of performing biophysical gene transfection with no additional equipment or EP consumables: microcell electroporation (ME). Using material and expertise widely available to the average investigator at a total cost of < $100 CAD, a range of programmable EP experiments could be performed over varied field strengths, pulse widths and sequences for mammalian EP cell transfections, demonstrating improved efficiency compared to standard commercial EP systems. With this approach, for CRISPR-mediated gene modifications of ES cells, sufficient modified lines could routinely be derived from a single 5 μl electroporation event utilizing 25,000 cells. This implementation thus addresses cost issues associated with bulk EP in addition to enhancing transfection efficiency of sensitive, difficult to transfect cell types while maintaining the speed and ease of use issues inherent to EP.

## Results

**Figure 1:**
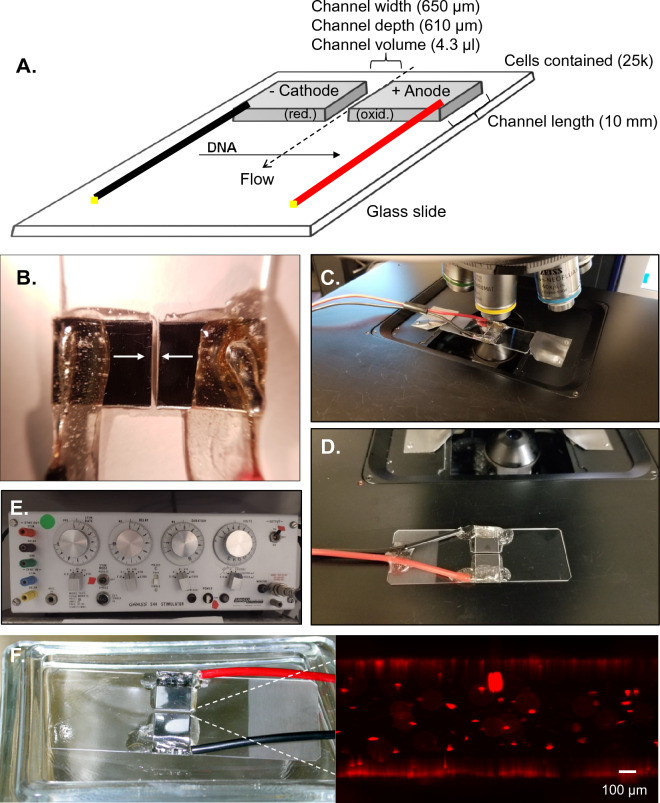
Microelectroporation cell. (**A**) Schematic overview of unit with most commonly utilized dimensions shown. Cell solution is confined in channel by hydrophobic forces. Direction of electrically induced DNA movement is indicated. (**B**) Polished 1 × 1 cm sections of 316 stainless steel separated by a gap distance of 650 μm (arrows) with connections soldered and protected using epoxy on a standard glass slide. (**C**) Reusable unit allowed rapid flushing and real-time inspection of electroporation on standard upright microscope. (**D**) Alternative attachment is shown. (**E**) Example of prefab electrophysiology unit which can be utilized for microelectroporation due to reduced gap size and fluidic volume. (**F**) Example of unit used for electroporation of ES cells, demonstrating development of propidium iodide-positive ES cells within well chamber within 2 min following application of pulsed electroporation. Scale bar represents 100 μm.

In order to optimize conditions for ME of difficult to transfect cells, we designed a durable, dynamic, low-cost electroporation chamber. As shown in Fig. 1A-B, polished 1 × 1 cm sections of 0.024″ thick 316 stainless steel were separated at fixed gap distances ranging from 200–1000 μm, serving as electrodes. Electrodes were then affixed and protected with epoxy on a standard glass slide (Fig. 1B, arrows)., While experiments were routinely performed in a class II-A2 biosafety cabinet with cell solutions confined within the electroporation channel, samples could be cover-slipped for real-time analysis on a standard upright or inverted microscope (Fig. 1C-D). Due to the reduced electrical requirements resulting from the diminished gap size (700 μm shown), electronics such as those commonly employed for electrophysiology are capable of supplying sufficient power for electroporation (Fig. 1E). In fact, further experimentation demonstrated that the electronics required could be wholly replaced by a battery powered Arduino based system with equal efficiency at a cost of < $100 CAD (Fig. 5). Using this system, relative cell permeability was assessed by monitoring the relative rate of fluorescence enhancement of cells to the cell-impermeant marker propidium iodide (PI). Under conditions appropriate for electroporation, a rapid increase in PI fluorescence could be observed in a substantial subpopulation of ES cells within 2 min of electroporation (Fig. 1F).

### Microelectroporation optimization

**Figure 2:**
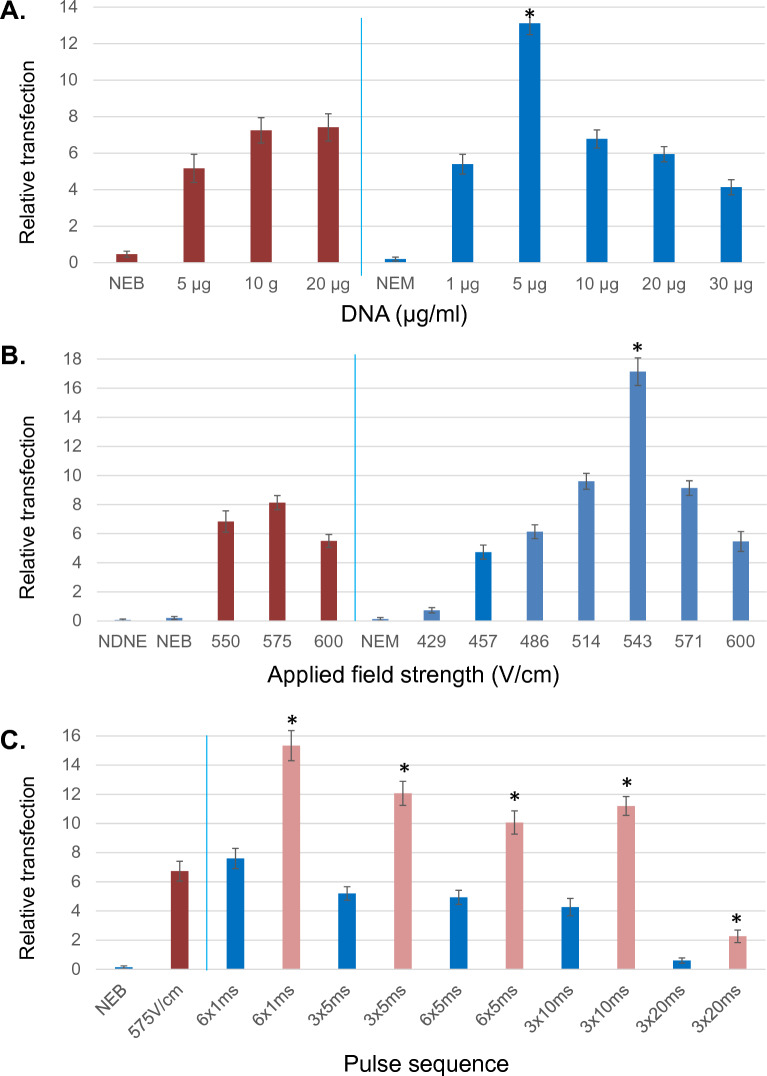
Optimization of microelectroporation. (**A**) Transfection was examined as a function of DNA reporter concentration; red-Bio-Rad gene pulser, blue-microelectroporator. NEB-no electroporation Bio-Rad, NEM-no electroporation microelectroporator. Experiments were performed in triplicate for each set with n > 4 sets for Bio-Rad conditions, n > 5 sets for microelectroporator. Field strength was set to 575 and 571 V/cm for Bio-Rad versus microelectroporator respectively. Capacitance for Bio-Rad was set to 500 μF and (τ) = 6.5 ms. Pulse sequence used for microelectroporator was 6 × 1 ms. *- Denotes significant enhancement at *P* < 0.01 over all Bio-Rad conditions. (**B**) Relative transfection efficiency as a function of applied field strength; red-Bio-Rad gene pulser, blue-microelectroporator. NDNE-no DNA no electroporation. Experiments were performed in triplicate for each set with n > 4 sets for Bio-Rad conditions, n > 5 sets for microelectroporator. DNA reporter set to 10 μg/ml for both systems. Capacitance for Bio-Rad was set to 500 μF and (τ) = 6.5 ms. Pulse sequence used for microelectroporator was 6 × 1 ms. Cells plated at 10 k cells/well in a 6 well. *- Denotes significant enhancement at *P* < 0.01 over all Bio-Rad conditions. (**C**) Relative transfection efficiency as a function of square-wave pulse; red-Bio-Rad gene pulser, blue -microelectroporator (plating: 10 k cells/well, 6 well plate), pink-microelectroporator (plating: 50 k cells/well, 6 well plate). Experiments were performed in triplicate for each set with n = 5 sets for both systems. DNA reporter set to 10 μg/ml for both systems, field strength was set to 575 and 571 V/cm respectively for Bio-Rad versus microelectroporator Capacitance for Bio-Rad was set to 500 μF and (τ) = 6.5 ms. *- Denotes significant enhancement at *P* < 0.01 over lower plating density (10 k cells/well).

We next optimized conditions for electroporation of embryonic stem cells using ME. To determine the true efficiency of the system, attempts were made to vary one critical parameter at a time. Results were assessed as a function of viable puromycin resistant colonies recovered following the electroporation of a 14.8 kb plasmid (Addgene #52961) and subsequent puromycin selection. Doing so allowed not only assessment of faithful plasmid uptake and expression, but also resulting ES cell survival, morphology and growth. As shown in Fig. 2A, results were compared between Bio-Rad Gene Pulser with Capacitance Extender and ME using equivalent initial electroporation (5,000 cells/μl), and plating (10,000 cells/well) cell concentrations. Resuspension buffer (EmbryoMax electroporation media) and field strengths (575 versus 571 V/cm) were also kept comparable. Additional set point conditions for Bio-Rad were 800 μl of cell solution in a 4 mm cuvette, absolute voltage 230 V, 500 μF, time constant (τ) = 6.5 ms. For ME values were: 4.3 μl sample volume, voltage 38 V for a gap distance of 700 μm. Based on the Bio-Rad time constant (τ), square-wave pulse sequence was 6 × 1 ms. Changes in DNA reporter concentration from 5–20 μg/ml resulted in consistent and relatively minor alterations in transfection efficiency for the Bio-Rad system (Fig. 2A). By comparison, alterations over this range for the ME resulted in significant alteration of the numbers of puromycin resistant colonies observed, with 5 μg/ml determined to be optimal compared to all other concentrations tested (Fig. 2A). Transfection efficiency as a function of field strength is shown in Fig. 2B, demonstrating a maxima at 575 V/cm for Bio-Rad system and 543 V/cm (applied voltage: 38 V) for ME among the conditions examined. Additional set point parameters for both systems are as given above for Fig. 2A, with the exception that DNA reporter addition was set to 10 μg/ml for both units. The results demonstrate that despite similar field strength maxima, ME demonstrated significantly higher transfection rates at its optimum field strength.

A parameter previously shown to significantly affect transfection efficiency is the nature of the square-wave pulse provided. To this end, the efficiency of different pulse wave parameters were examined, with DNA reporter addition set to 10 μg/ml for both systems and field strength set to 575 and 571 V/cm for Bio-Rad and ME respectively. Modification of pulse length to either longer time periods, or greater pulse number did not enhance overall ME transfection efficiency beyond that seen at 6 × 1 ms (Fig. 2C). Thus one millisecond pulses appear capable of generating pores of sufficient size and longevity to allow the uptake of a 14 kb plasmid to occur. However given that embryonic stem cells are known to exhibit significant density dependence with respect to cell survival, it is possible that a portion of transfected cell die subsequently, an effect which might be rescued by enhancing cell density. As such the efficiency of transfection was examined as a function of plating density. As shown in Fig. 2C, increasing plating density from 1,042 cells/cm^2^ (10 k cells/well of 6-well plate) to 5,208 cells/cm^2^, demonstrated significant enhancement in relative transfection at higher plating density compared to lower plating density for all pulse parameters examined (all results presented corrected to 1042 cells/cm^2^).

**Figure 3:**
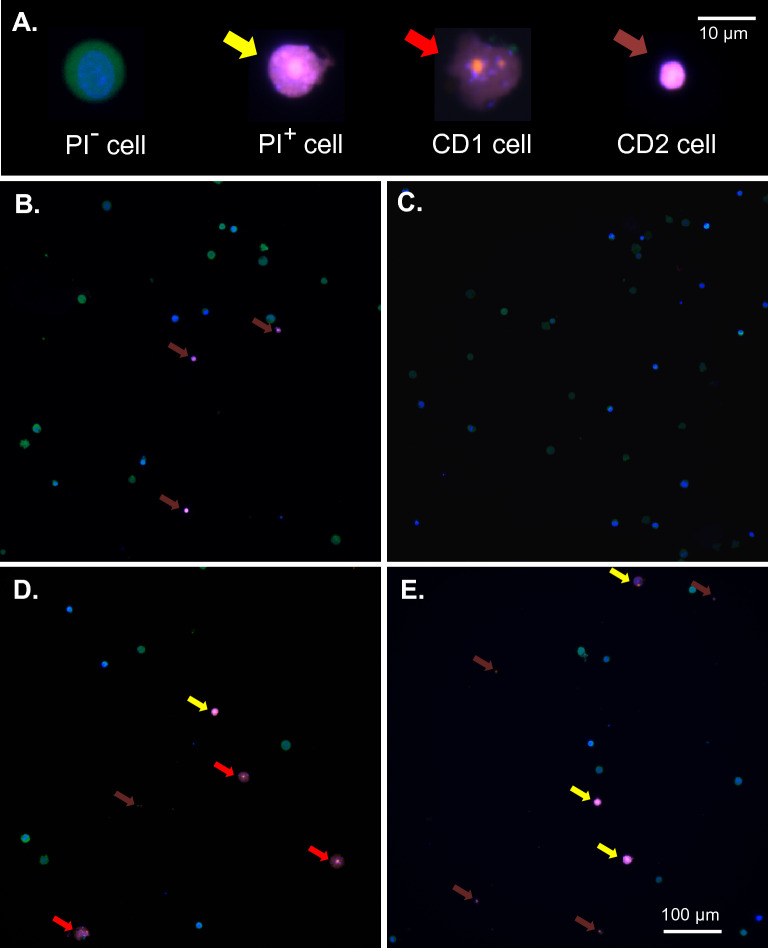
Electroporation isotypes. (**A**) Examples of the morphology of major ES cell isotypes observed before and following electroporation. CD1/2 = cell death type 1/2. Scale bar represents a distance of 10 μm. (**B**–**E**) Examples of pre- (**B**, **C**) and post- (**D**, **E**) electroporated average fields. Scale bar in (**E**) represents a distance of 100 μm. Where indicated cells are shown 10 min following electroporation (ME: 543 V/cm, 6 × 1 ms), in which citrine-expressing ES cells were pre-incubated with both the cell permeant marker Hoechst 33342 and cell impermeant marker propidium iodide. Cell types were broadly defined as: PI- cells displaying no membrane disruption or permeation of PI; PI+ cells exhibiting PI permeation without significant disruption of internal structure (yellow arrows); PCD1 cells displaying PI permeation with early collapse of internal structures (red arrows); PCD2 cells displaying PI permeation with complete collapse of cellular volume to < 50% of PI- cells (brown arrows).

Despite optimizing transfection efficiency in terms of DNA concentration, field strength and pulse sequence in ME, successful transfection with expression of plasmid-based targets is still only achieved in a minority of cells in difficult to transfect cell lines. One potential source of this inefficiency is the permeabilization characteristics of the targeted population. In order to better understand this process with respect to ME, ES cell morphology was examined as a function of electroporation conditions. Figure 3A demonstrates the major morphologic isotypes observed in the presence and absence of electroporation. For these experiments ES cells expressing monomeric cytoplasmic citrine from the *Rosa26* locus were utilized in conjunction with pre-incubation of both the cell permeant marker DNA marker Hoechst 33342 and cell impermeant marker propidium iodide. As shown in Fig. 3, under the most optimal ME transfection conditions examined (543 V/cm, 6 × 1 ms) cells unaffected by electroporation were frequent (PI^-^, 58 ± 4%, n = 300), exhibiting cytoplasmic citrine fluorescence with only Hoechst staining within the cell nucleus and lacking plasma membrane interruption with propidium iodide entry. By contrast, a minority of cells (PI^+^, 5 ± 2%) exhibited entry of propidium iodide in the period following electroporation (t = 10 min) without cellular collapse or significant disruption of internal structures (yellow arrow). More prevalent (cell death type 1, 14 ± 4%) were cells presenting PI permeation with early collapse of internal structures and nuclear blebbing (red arrow). Another common isotype (cell death type 2, 23 ± 3%) were cells displaying PI permeation with substantial structural collapse such that cellular volume was ≤ 50% of untransfected cells (brown arrow). Alteration of pulse sequences to longer time periods (5, 10 and 20 ms) increased relative numbers of cell death type 1/2 cells but did not increase numbers of ‘PI^+’^ cells, mirroring transfection effects (Fig. 2C). Therefore a more extended series of shorter (1 ms) electroporation pulses may be a more optimal approach to enhancing transfection efficiency.

To further examine the electroporation efficiency of both Bio-Rad and ME electroporators in cell lines recalcitrant to transfection, primary murine fibroblasts and human T lymphocyte Jurkat cells (clone E6-1) were examined at several conditions as shown in Supplemental Figure S1. For purposes of comparison, conditions were set to those previously reported as optimal for the Bio-Rad electroporator. For both cell types and ME and Bio-Rad electroporators, cell and DNA concentration were kept at a constant (3000 cells/μl, 20 μg/ml mRuby3/mClover3 fluorescent expression vector-Addgene #74252), despite these parameters being outside the observed optimum for ME electroporation of ES cells (5 versus 20 μg/ml, Fig. 2A). With respect to primary fibroblasts, Bio-Rad and ME electroporators exhibited similar efficiencies at their observed optimums (Bio-Rad-800 V/cm, 4 mm cuvette, τ = 14.5 ms; ME- 6 × 1 ms). By contrast, for Jurkat cells (Supplemental Figure S1, S2), ME electroporation exhibited significant enhancement over Bio-Rad at its observed optimum (Bio-Rad-800 V/cm, 4 mm cuvette, τ = 16.5 ms; ME-6 × 1 ms). In both cell types under these conditions the observed maxima for ME electroporation conditions appeared at lower field strengths compared to that seen with Bio-Rad. Example fields for both primary fibroblasts, and Jurkat cells are shown for ME and Bio-Rad mediated transfection respectively (Supplemental Figure S1). Interestingly for both cell types, ME-transfected cells routinely exhibited greater fluorescence intensity compared to their Bio-Rad transfected counterparts under optimal conditions.

### CRISPR-mediated gene modification using ME

**Figure 4:**
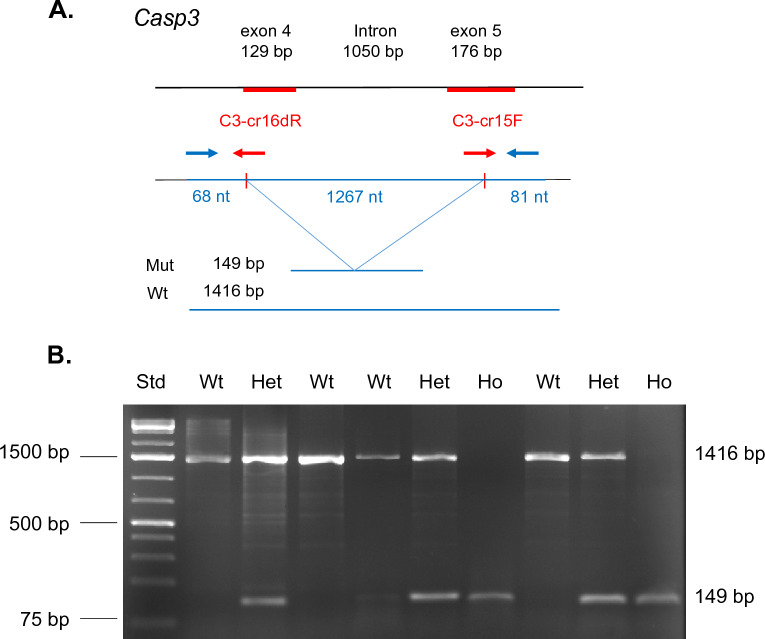
Use of microelectroporation for CRISPR mediated gene editing. (**A**) Example of stratagem employed for modification of Casp3 locus. Combined action of cloned CRISPR single guides (red arrow) results in a deletion of 1267 nucleotide resulting in a frameshift mutation between exons 3 and 4. In such an event PCR primers (blue arrows) identify a band of 149 bp for the mutant allele versus 1416 bp for wildtype (unmodified) locus. (**B**) DNA electrophoresis of 9 puromycin-resistant ES cell clones derived following microelectroporation. Examples of wildtype (Wt), heterozygous (He), and homozygous (Ho) modifications are observed. DNA molecular weight standard (Std.) is shown at left.

Given that a primary motivating factor for designing the ME system was the performance of CRISPR-mediated gene targeting in embryonic stem cells, we assessed the ability of ME to produce targeted mutations for several loci of interest. An example of this is shown in Fig. 4A, using multiple CRISPR single guide RNAs (sgRNA) targeting exons 4 and 5 of the *Casp3* gene. To create sgRNA expressing CRISPR plasmids, CRISPR RNA (crRNA) sequences were cloned into a tracrRNA containing Cas9 expression vector (Addgene #52961). CRISPR sgRNA plasmids were then electroporated at a final concentration of 5 μg/ml (total) with ES cells (5,000 cells/μl) in ~ 5μλ at 543 V/cm (6 × 1 ms) in EmbryoMax electroporation media. Following electroporation, cells were plated at 10,000 cells/well in 6-well dishes and subjected to puromycin selection on days 2, 3, and 5. Five days following antibiotic removal, puromycin-resistant ES colonies were counted, replica plated and analyzed. As shown in Fig. 4B, successful deletion employing both CRISPR sgRNA plasmids would result in a reproducible deletion of a segment of exon 4, exon 5 and the intervening intron, removing a ~ 1267 nucleotide segment. PCR primers utilized for this analysis are shown (blue arrows, Supplementary Table S1).

Using the selection protocol outlined above, we examined the ability of the ME electroporator to produce defined biallelic gene deletions in several target genes of interest via the simultaneous introduction of plasmids encoding CRISPR sgRNAs. The sequence and details of these targeting events are shown in Supplemental Figure S3, together with the genomic deletions induced as determined by direct sequencing of clones. The crRNA sequences used to generate plasmid-encoded CRISPR sgRNAs are shown in Supplemental Table S2. Target sequences for genes of interest were obtained from Ensemble and verified via Sanger sequencing of ES cells for the genetic background of interest (129/Sv). In each case, experimental sequences were identical to those predicted for CRISPR targets despite different genetic backgrounds (C57bl/6 J versus 129/Sv). For each gene locus examined, CRISPR sgRNA plasmid pairs were successful in producing biallelic deletions based on Sanger sequencing of the clone populations obtained (Supplemental Figure S3). Conveniently, such method of targeting could be readily identified by standard PCR. The most common deviation observed in this targeting was that several CRISPRs cleaved 1–3 nucleotides up/downstream of the canonical cleavage site (between nucleotides 3 / 4 upstream of the PAM site). Consistent with prior observations^34^, CRISPRs varied in their ability to produce biallelic deletions at different genetic loci (**caspase-3** WT 48% He 39%, Ho 13%; **caspase-8** WT 37%, He 32%, Ho 31%; **MLKL** WT 23%, He 69%, Ho 8%-of puromycin resistant clones). Despite such differences, none of these frequencies fell below numbers readily obtainable following one ME selection event (> 50 puromycin resistant clones).

### Development of microcontroller-based ME wave generator

Having validated the operation of the ME chamber (see Methods), we sought to further improve the flexibility, portability and accessibility of ME by re-engineering its electrical aspects. As a reduction in electroporation volume lowers capacitance requirements, standard electrophysiology stimulators such as that shown in Fig. 1E (Grass S44) could be utilized to generate the frequency, magnitude and duration of waveforms required. However given recent advances and cost reductions in microprocessers, such requirements could also be met by circuitry supported by Arduino-based microcontrollers. We therefore designed the layout of a small scale Arduino-based electroporator, whose capabilities were well within the waveform, voltage and frequency requirements of ME. Transfection experiments comparing the performance of Grass S44 versus Arduino-based electroporator demonstrated no differences. However the Arduino-based microcontrollers significantly reduced the total cost and size of the electroporation unit while maintaining flexibility of waveform generation. Through provision of a detailed parts list, schematics and instructions (see below and Supplemental Figs. 4–5), even those without detailed electronics experience can readily construct an ME electroporator.

**Figure 5:**
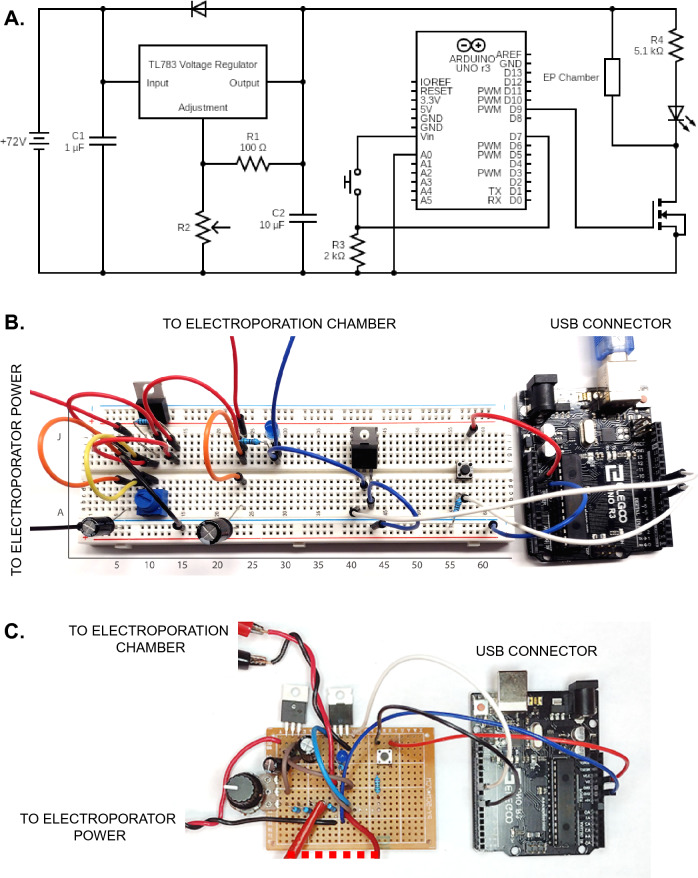
Electroporator schematic and practical examples. (**A**) Electrical schematic of Arduino based electroporator. (**B**) Visual example of electroporator assembled on breadboard as indicated, or (**C**) soldered on a protoboard. Connections from wires to other non-visible components are indicated.

### Construction of microcontroller-based ME wave generator

Electroporator parts list. Note**: Bolded items** were obtained from Arduino starter kit (ELEGOO, EL-KIT-003) but may also be acquired separately. Sources indicated for each component. Due to their low cost, extras of individual components are recommended as backup. Individual components can be obtained from local or online electronics distributors such as Mouser Electronics and Digi-key Electronics, or Amazon for more common items.

- **1 × Arduino Uno R3 and USB cable**
- **1 × Breadboard (830 tie points)**
- **1 × 4 pin momentary push button**
- **17 × Male to male jumper wires**
- **1 × each 100 Ω, 2 kΩ, 5.1 kΩ resistors**
- **1 × Light emitting diode (LED, through-hole)**
- 1 × Adjustable linear voltage regulator (Texas Instruments, TL783CKCSE3).
- 1 × N-channel MOSFET (Infineon, IRF540NPBF).
- 1 × each Capacitors (1 μF and 10 μF) (Nichicon, UVZ2C100MPD, UVZ2C010MED).
- 1 × Diode (ON Semiconductors, 1N4003G).
- 1 × 10 kΩ Potentiometer/Adjustable resistor (Bourns, 3386P-1-103 T).
- 2 × Alligator clips and wires (eBoot, EBOOT-TEST-LEAD-01).
- 8 × 9 V Batteries (Amazon Basics, 6LR1).

As common breadboarding issues involve misaligned or reversed components, an exact position for each component on a standard breadboard is indicated below and shown visually in Figure S2. Components for which directionality is important are identified in Figure S1 and orientation specified below.

Component connections to specific breadboard pins:

TL783 voltage regulator: J10, J11, J12-Adjustment (A), Output (O) and Input (I) pins respectively
IRF540N MOSFET: E41, E42, E43-Gate (G), Drain (D), Source (S) pins respectively
Push button: F56, F58, E56, E58
Diode: Cathode (side with grey stripe) G7, anode G11
Light emitting diode: Cathode (short leg) I29, anode (long leg) I28
Capacitor 1 (1 μF): Cathode (short leg) GND rail, anode (long leg) A7
Capacitor 2 (10 μF): Cathode (short leg) GND rail, anode (long leg) A24
R1 (100Ω): I10, I14
Potentiometer (R2): B10, A11/B11, B12-terminal 1, wiper, terminal 2 pins respectively (see note below)
R3 (2 kΩ): B56, GND rail
R4 (5.1 kΩ): G24, G28
Jumper wires: F7 to E7
  F10 to E10
  E11 to GND rail
  H7 to H12
  F11 to F14
  H14 to H24
  F24 to E24
  F29 to D42
  A41 to Arduino pin D9
  A43 to GND rail
  C56 to Arduino pin D7
  J58 to Arduino 5 V pin
  Arduino GND pin to GND rail
Open jumper wires: J7 (for positive battery terminal connection)
  GND rail (for negative battery terminal connection)
  J24 (for positive terminal of ME chamber connection)
  G29 (for negative terminal of ME chamber connection).

*Additional construction notes*: The potentiometer listed in the parts list has the wiper pin offset downwards by one position. Thus it would be attached to position “A11” rather than “B11” (as shown in Figure S2). Open jumper wires from circuit to batteries and ME chamber can be connected via alligator clips or by other means as needed. The Arduino microcontroller can be powered by computer via supplied USB cable or a separate 9 V battery via barrel jack. Output voltage is determined by the equation V ≈ 1.25 x (1 + R2/R1), where R1 is typically a fixed value (we utilize a 100 Ω resistor), with variable R2 resistances (fixed values or variable potentiometer-see below). A standard multimeter can be used to verify R2 resistance or to measure voltage across the electroporation chamber electrodes for greater precision. In place of a potentiometer, fixed resistors for R2 can be used for applications where variable output voltage is not required. Note that theoretical vs. actual voltage output may differ and measurements for the system used in this study are indicated in Figure S2.

*Arduino enabling*: Software required to communicate with the Arduino (Arduino IDE) is available at https://www.arduino.cc/en/software, which is used to modify and upload code via USB cable (see Figure S2).

*Waveform setup* The Arduino microcontroller controls pulse duration with several lines of code. The entirety of the code is shown in Figure S2, and can be downloaded from the supplemental data (Momentary_Switch_for_Electroporation.ino) or at db.phm.utoronto.ca/ME.htm. Once uploaded to the Arduino, pressing the push button will result in a single 1 ms pulse to the ME chamber. Pulse duration may be altered by adjusting the source code as shown in Figure S2, specifically by altering the value of “pulseDura” (assumed value in milliseconds) in the source code. Note that upon alteration of any source code, the code must be reuploaded to the Arduino for these changes to take effect. For modifications of pulse duration to values of < 1 ms, change the “delay(pulseDura)” command to “delayMicroseconds(pulseDura)”. This will alter the program to generate a pulse with duration equal to the value of “pulseDura” in microseconds instead of milliseconds.

*Circuit testing*: For internal verification, the LED will briefly flash once when the push button is depressed, serving as an indicator of power reaching the EP chamber. Changes in output voltage and pulse duration will also be reflected in the intensity and duration of the LED pulse. Direct confirmation of waveform characteristics can additionally be determined using an oscilloscope as shown in Figure S2. Most aspects of the circuit (save absolute output voltage) can be tested using a single 9 V battery for safety.

## Discussion

Electroporation has been used for forty years as a means of cell transfection^35^. However recent advances in the efficiency of genome editing using techniques such as CRISPR have provided new opportunities for such modifications in even difficult to transfect cells. We describe results obtained using a novel ME method as an efficient cost-effective alternative to conventional bulk electroporation. Using this simple scaled down system, we were able to drastically reduce equipment and reagent costs while simultaneously improving efficiency and portability. Consequently, ME could achieve results using just a fraction of the cells typically required, in volumes of a few microliters, making it suitable for studies employing specialized cell types and/or reagents where standard volumes may impose a barrier; particularly at scale. One immediate effect of this is to dramatically reduce the cost of such experiments due to lowered reagent requirements across the board. In keeping with this, the ME chamber is constructed for repeated use with cleansing and sterilization by 70% ethanol and as such does not pose an ongoing consumable cost as single use cuvettes do. Due to their low surface area, EP chamber faces could easily be electroplated with additional metals such as gold or platinum to protect against corrosion, however based upon results obtained over a period of several years we observed no need for such modifications in terms of performance. This is likely due to both the nature of the molybdenum marine grade 316 stainless steel and the relatively short time frame (1–2 min) of exposure to saline solutions. By contrast, most commercial electroporators utilize disposable single-use cuvettes and/or proprietary reagents for which costs can be significant over time. Similarly, other comparable methods of cell transfection such as lipofection require no equipment but pose much higher reagent costs presenting a challenge; particularly as scale.

While the method described allows for construction of a variety of electroporation chambers, with respect to practical utility and ease of use, volumes of 4–5 μl (650–700 μm gap distances) appeared optimal for most projects. In part this was dictated by numbers of genetically modified cells required to confidently obtain multiple cell lines containing biallelic modifications of the target gene of interest. However an additional factor when considering anode/cathode distances is examined by Li et al., who explored electric field distributions at gap distances of 100, 200 and 500 μm; demonstrating the resulting field is significantly less uniform at smaller distances compared to 500 μm or greater^31^. The authors also examined the effect of electroporation-induced pH fluctuation at anodes and cathodes, noting enhanced cellular injury at small gap distances (100–200 μm), though these studies examined higher electric field strengths (800–900 vs. 543 V/cm), greater numbers of applied pulses (20 vs. 6), and extended duration within the EP chamber (10–60 min. vs. 1 min.) compared to the current study. Despite this, electroporation-induced pH fluctuation is a significant consideration as the authors demonstrated rapid induction of cell death at pH below 4 or above 10^31^. In keeping with this, the chamber used for current study (650 μm gap distance) demonstrated anode/cathode pH values of 6.5 and 7.6 respectively using the described electroporation buffer, with cells typically diluted 200-fold in growth media immediately following electroporation. Furthermore, due to the large surface area to volume ratio of the chamber, cell injury due to thermal heating is not a major consideration in this design.

Efficient transfection has been shown to critically depend upon maintaining proper balance between pore number and size within the cell membrane versus levels of overt cellular destruction^36,37^. As demonstrated theoretically and experimentally by the studies of Saulis and Saulė, for square wave pulses of 0.1–2 ms at field strengths of 0.2–2.4 kV/cm, membrane pore size increases with longer pulse period^38^. Increasing pore size not only allows transport of larger molecules natively, but further supports this process through the presence of longer recovery periods. Thus millisecond pulses generate larger pores than microsecond pulses and exhibit longer recovery times (on the order of 10–20 min) even in the absence of cell cooling below the membrane transition temperature^37,38^. Additionally, the work of Pucihar et al. using Chinese hamster ovary (CHO) cells examined properties of pulse equivalence, specifically, conditions required to successfully electroporate similar fractions of cells at different pulse durations, demonstrating lower field strength are required at longer pulse periods^36^. Their determined value of ~ 450 V/cm for pulse periods of 1 ms in CHO cells agrees well with experimental values observed for ES cells considering their proportionally smaller cell volume. Finally with respect to optimizing EP waveform, the work of Liu and Bergan demonstrated square wave electroporation to be more effective than exponential decay for the introduction of oligonucleotides in primary (hematopoietic) cells^39^.

Development of simplified electroporators is not new, with several groups previously describing the potential of such devices to promote the transfection of bacterial cells^40–42^. Given this issue has already been well addressed by others, and the presence of additional straightforward options such as heat shock to perform bacterial cell transfection, we have focused our efforts on difficult to transfect mammalian cells. While the maximum field strength of the system was not determined, we observed that using a 72 V supply with a 0.65 mm ME chamber, electric fields of > 950 V/cm could easily be generated; well beyond the optimum for all mammalian cell lines tested in the millisecond pulse range. The current device requires no complicated manufacturing processes such as PDMS microchip fabrication, photolithography or 3D printing, can be constructed by a novice in minimal time (1 h) without the use of specialized tools and utilizes materials readily available to the average investigator from numerous sources. At a unit cost of < $100 CAD it provides a simple, portable (130 g plus eight 9 V batteries weighing 430 g total), efficient open source electroporator, containing explicit circuits, code, parts list and step by step manufacturing instructions for the ME chamber and associated waveform generator. Transparency of the ME chamber allows real-time brightfield and fluorescent imaging measurements. Given enough supply power and using the described ME chamber, the current ME electroporator is adjustable to field strengths of at least 1.4 kV/cm without any modification to existing circuitry. The aim of the current instrument is to address the need for an intermediate between traditional bulk electroporation and single cell approaches. The described instrument is capable of providing sufficient transfection capacity to enable current generations of gene modification work for developing stable genetically modified cell lines using tools such as CRISPR and base editors. With respect to such approaches, the small chamber capacity and enhanced efficiency allows ~ 500 fold reduction in typical required protein, oligonucleotide and mRNA requirements, resulting in significant cost savings and more optimal reagent concentrations compared to traditional EP. Such design also provides an opportunity to perform EP experiments on limited populations or difficult to obtain cells. While additional factors which may further enhance efficiency, such as cell cooling following electroporation, were not systematically explored in the current study, it is hoped that the open source format will provide researchers the ability to pursue their own specialized interests.

## Methods

### Plasmid creation and preparation

DNA sequences encoding investigator designed gRNAs (Supplemental Table S1) were inserted into lentiCRISPRV2 plasmid (Addgene #52961) or px459v2 (Addgene # 62988) encoding for guides scaffolds, nuclearly localized Cas9 protein and Puromycin resistance gene linked by P2A sequence. pKanCMV-mClover3-mRuby3 (Addgene #74252), EGFP-puro (Addgene #45561) and pEGFP-N1 (GenBank accession #U55762) were also utilized as transfection controls plasmids and for quantification. Plasmids were prepared from transfected bacterial culture stock grown overnight in ampicillin containing LB^43^ at 37 °C at 250 rpm. The following day plasmids were isolated using QIAprep Spin Miniprep Kit (Qiagen 27104) and EndoFree Plasmid Maxi Kit (Qiagen, #12362), to acquire purified DNA with low bacterial endotoxin. Plasmid integrity was verified using gel electrophoresis and transfection quality was independently determined using lipofectamine as previously described^44^.

### Cell culture and transfection

Primary murine embryonic stem cells were generously supplied by the laboratory of Dr. M.A. Magnusen, Vanderbilt University^45^ and were maintained at 37 °C and 6% CO_2_ in media containing high glucose DMEM (Invitrogen 11960–044), 2 mM L-glutamine (Invitrogen 25030), 2 mM GlutaMAX (Invitrogen 35050), 0.1 mM 2-mercaptoethanol (Sigma M7522), 0.1 mM MEM non-essential amino acid (Invitrogen 11140), 1 mM sodium pyruvate (Invitrogen 11360), 50 U/ml penicillin/streptomycin (Invitrogen 15140), 1000 U/ml LIF (Chemicon ESG1107), and 15% ES cell qualified fetal bovine serum (FBS). Primary murine fibroblasts were derived locally from CD-1 mice as previously described^46^ and cultured in high glucose DMEM supplemented with 10% FBS, 2 mM glutamine and 100 U/mL penicillin/streptomycin. Human T lymphocyte Jurkat cells (Clone E6-1, ATCC TIB-152) were cultured similarly in RPMI-1640 (Sigma R8758) with 10% FBS, 0.1 mM 2-mercaptoethanol, and 100 U/mL penicillin/streptomycin. ES cells were grown on multidrug resistant DR4 mitomycin-treated fibroblasts. ES cells were used for ME at > 60% confluency. Media was routinely changed 2 h prior to electroporation. ES cells were then trypsinized, centrifuged at 300 × g and resuspended in growth media (fibroblasts and Jurkat cells resuspended directly into EmbryoMax for electroporation). ES cells were plated on gelatin-coated plates for 30 min to remove associated mitomycin-treated fibroblast. Media containing purified ES cells was then centrifuged to pellet cells and then cells were gently resuspended in EmbryoMax electroporation media (Millipore ES-003-D, 4.5 g/l glucose, 2.3 g/l bicarbonate, pH 7.3, 327 mOsm) and diluted to a concentration of 5000 cells/μl (ES cells) or 3000 cells/μl (fibroblasts, Jurkat cells) for ME. ES cells were then transfected according to the parameters indicated (see Results) and plated onto mitomycin-treated DR4 fibroblasts at a concentration of 10 k-50 k cells/well of a 6-well dish where indicated and allowed to recover. Twenty-four hours following electroporation (day 0–1) ES cells were subjected to puromycin (BioShop, PUR333.100) selection for 5 days (given on days 2, 3 and 5) at concentrations titrated to be to 100% lethal for each cell line. Following selection, numbers of surviving transfected clones were then determined for each plate using light and fluorescent microscopy. Fibroblasts and Jurkat cells were returned to respective growth media for 1–2 days and transfection efficiency was determined as a percentage of total population with fluorescent microscopy.

### Cell line genotype analysis

For those colonies subjected to genotyping, individual ES colonies were picked and placed into replicate wells of a 96-well plate. At sufficient density cells were lysed in 50 μl lysis buffer/well (100 mM Tris–HCl (pH 8.0), 5 mM EDTA, 0.2% SDS, 200 mM NaCl, 100 μg/ml Proteinase K) overnight at 37 °C in a humidified chamber. The following day 100 μl of ice cold NaCl/ethanol mixture (1.5% 5 M NaCl v/v in 100% ethanol) was added to each well and the plate was allowed to sit for 30 min at -20 °C. Plates were then carefully inverted to remove excess liquid. Wells were then carefully washed with 100 μl of 70% ethanol and inverted to remove the liquid. This was repeated three times and samples were left to allow ethanol to evaporate. DNA in each well was then resuspended in 40 μl of 10 mM Tris pH 8. PCR was then performed using primers appropriate for targeted genomic regions (Supplemental Table S2). PCR products were run on a 2% agarose gel stained with 5 μl Midori Green Advance DNA Stain (Nippon Genetics Europe MG04) per 100 ml of gel to visualize at 540 nm and used for Sanger sequencing.

### Fluorescent imaging

Cell imaging of ES cells for purposes of quantifying pre- and post-transfection cell structure and efficiency were recorded at 20 and 40 × using on a Nikon E1000R microscope equipped with standard DAPI, FITC, TRITC and Cy5/DiD excitation/emission filters. Fluorescent cell counts were determined from images by manual inspection using Adobe Photoshop. Fluorescent images for quantification of Jurkat cells and morphological analyses of ES cells were captured using Zeiss AxioImager upright microscope with sCMOS camera and Zeiss ZEN software (blue edition, version 3.4.91.00000, available at www.zeiss.com/microscopy/en/products/software/zeiss-zen.html) at 20 × and 10 × magnification. Imaging of fibroblasts for quantification was performed using an Agilent Cytation 5 Cell Imaging Multimode Reader with DAPI, GFP, and Texas Red excitation/emission filters. Hoechst 33242 and PI were used at final concentrations of 2 and 1 μg/ml respectively.

### ME chamber construction

Micro-electroporation chambers were constructed using standard glass microscope slides as the base. Two 1 cm × 1 cm × 610 μm (0.024″) 316 corrosion resistant stainless steel sections (McMaster-Carr 88885K71) were cut using a metal shear press and optically flattened between metal plates. Chamber faces of each section was then polished with 800 grit silicon carbide sand paper, followed by one micron 3 M lapping film (BestSharpeningStones). 18 gauge wire was then soldered to the distal edge of the steel sections creating anode/cathode inputs. Distal ends of these wires terminated in alligator clips or gold pin connectors for attachment to commercial or constructed electroporators. Stainless steel sections were then placed onto microscope slides to create a channel of the desired width (200–1000 μm, 600–700 μm typical) parallel to the long axis of the slide. Sections were epoxied (Gorilla Epoxy, Cincinnati, Ohio) and clamped in place. Epoxy was also utilized to protect anode/cathode wire junction points and seal electrode elements around the chamber to confine the primary electrode channel (Fig. 1B). To ensure proper anode/cathode gap dimensions several coverslips of a defined thickness can be inserted between plates, however this was typically found to be unnecessary. Following curing, any excess epoxy was removed to create an unobstructed channel, resulting in the ME chamber shown in Fig. 1. Final chamber dimensions were then verified under a microscope using an ocular micrometer. Between samples and experiments, the ME chamber was thoroughly rinsed with sterile water and 70% ethanol. As required, electrode surfaces were cleaned with a solution of 0.8 M sodium bicarbonate using a nylon (tooth) brush, rinsed with water and dried with compressed air.

### Statistics

Statistical comparison between individual groups were performed using Student’s t test (unpaired, two tailed with assumption of equal variance) for examination of significance, determined at a minimum level of *P* < 0.05. Statistical analyses of greater than 2 groups with one independent variable were performed using one-way ANOVA and Tukey’s post hoc with significance defined at a minimum level of *P* < 0.05. Statistical significance between groups was determined using the non-parametric Mann–Whitney U-test with *P* < 0.01 considered to indicate significant differences. Statistical measures were performed using Microsoft Excel and GraphPad Prism Software, version 6. All values are presented as mean values ± SE.

### Supplementary Information

Supplementary Information 1.

Supplementary Information 2.

## Data Availability

Data generated or analysed during this study are included in this published article, supplementary information files or available upon request from the corresponding author (Jeffrey T. Henderson, jeff.henderson@utoronto.ca).
