# Supplementary material for: An efficient low cost means of biophysical gene transfection in primary cells
Source: Sci Rep. 2024 Jun 8;14:13179. doi: 10.1038/s41598-024-62996-y (PMC11161637; doi:10.1038/s41598-024-62996-y)
Supplement: Supplementary file 1 — Supplementary Information 1. [file 41598_2024_62996_MOESM1_ESM.pdf]

Supplemental Data

PCR primers	Sequence	Tm
Caspase-3 forward primer	AGCTCTCTCTCTGTCCCA	60°C
Caspase-3 reverse primer	TAACCAAACTGAGCACCGAG	
Caspase-8 forward primer	GCCCTCAAGTTCCTGTGCTT	60.5°C
Caspase-8 reverse primer	CAGCCTTTCTACCCACCTGT	
MLKL forward primer	GGACAGATCATCAAGTTAGGCC	60°C
MLKL reverse primer	TCTTATCATTGCCCACACTCAC	

Supplemental Table S1. Diagnostic PCR primers. Sequences and melting temperatures of PCR primers used for amplification of regions targeted for CRISPR modification.

Target	CRISPR crRNA ID	Sequence
Caspase-3	C3-cr16dR	CACCGGCCCATGAATGTCTCTCTG
	C3-cr15F	CACCGTCTTCAGAGGCGACTACTGC
Caspase-8	C8-cr1R	CACCGTAGCTTCTGGGCATCCTCGA
	C8-cr4R	CACCGCAGGTCCCACCGACTGATG
MLKL	MLKL-cr1R	CACCGCACACGGTTTCCTAGACGC
	MLKL-cr2R	CACCGACTTCATCAAAACGGCCCA

Supplemental Table S2. CRISPR RNAs. Sequences of crRNAs inserted into lentiCRISPR v2 plasmids used for CRISPR modification. Guide RNA scaffold (tracrRNA) from lentiCRISPR v2 is shown below. BsmBI cleavage sites were used to remove filler nucleotides into which crRNA sequences were inserted.

Supplemental Table S2 - Addendum

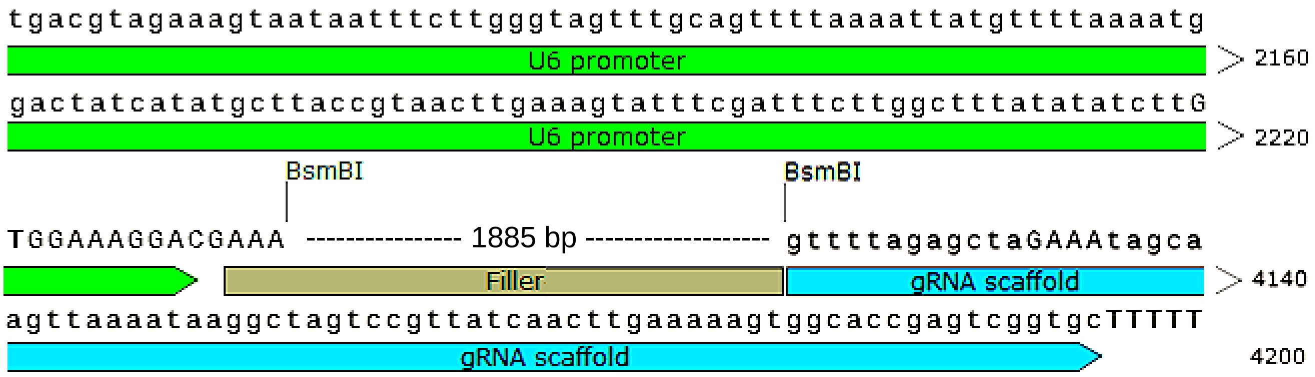

Supplemental Figure S1.

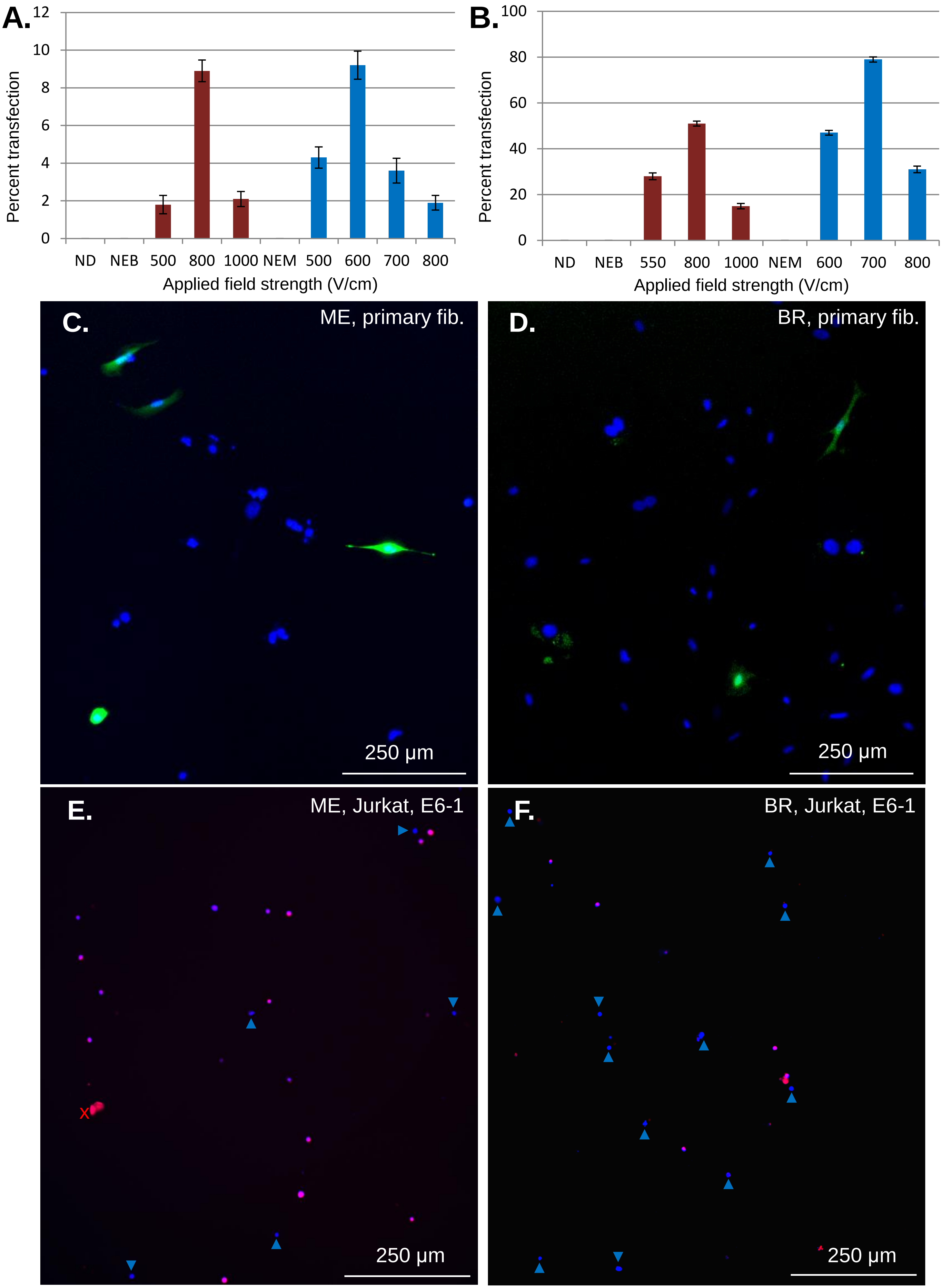

Supplemental Figure S1. Comparative electroporation efficiency in additional cell types. (**A, B**) The relative efficiency of Bio-Rad Gene Pulser with capacitance extender and ME electroporators were further examined at several field strengths for both primary murine fibroblasts (A), and immortalized Jurkat human T cells (clone E6-1) (B). * - indicates statistical significance at $p<0.05$ compared to optimal Bio-Rad conditions. (**C, D**) Examples of primary fibroblasts transfected via ME and Bio-Rad meditated electroporation respectively (BR - 800 V/cm, 4 mm cuvette, τ = 14.5 ms; ME – 600 V/cm, 6x 1 ms). (**E, F**) Jurkat cells transfected by transfected by ME and BR-meditated electroporation respectively (BR - 800 V/cm, 4 mm cuvette, τ = 16.5 ms; ME – 700 V/cm, 6x 1 ms). Due to their small cross-sectional area and to more readily distinguish transfected from non-transfected cells in fields (E) and (F), blue arrowheads denote those cells which do not express the fluorescent marker mRuby3, individual channels shown in Supplemental Figure 6. Scale bars denote a distance of 250 μm.

Supplemental Figure S2.

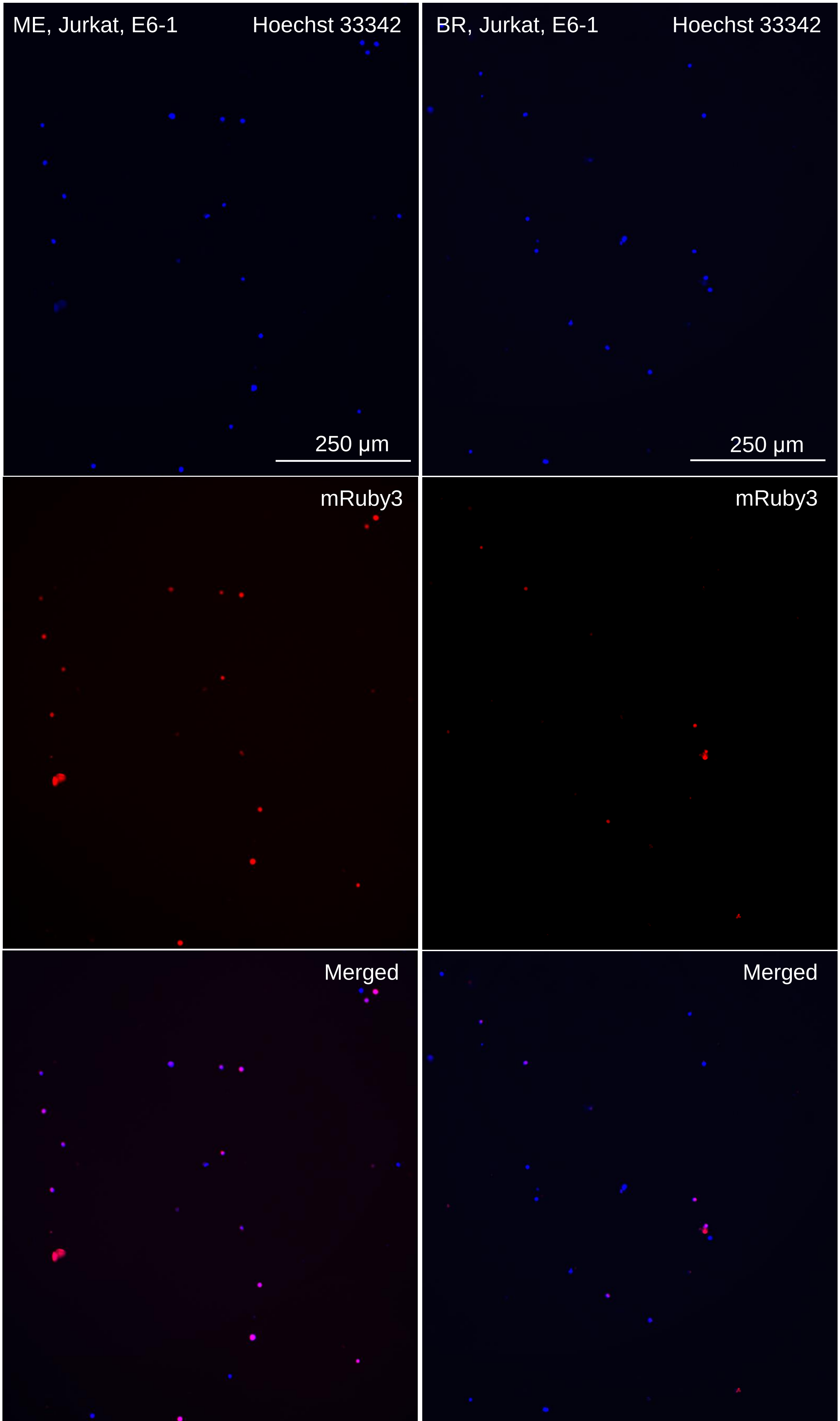

Supplemental Figure S2. Isolated and merged optical channels for Jurkat cells transfected via ME and Bio-Rad mediated electroporation. Cell were transfected with 20 µg/ml mRuby3/mClover3 fluorescent expression vector (Addgene #74252) analyzed 24 hours post-transfection following 10 minute incubation with 2 µg/ml Hoechst 33242. (**A**) Blue/Hoechst 33342 channel, (**B**) Red/mRuby3 channel, (**C**) merged layers. Scale bars denote a distance of 250 µm for all figures.

Supplemental Figure S3.

A.

Caspase-3

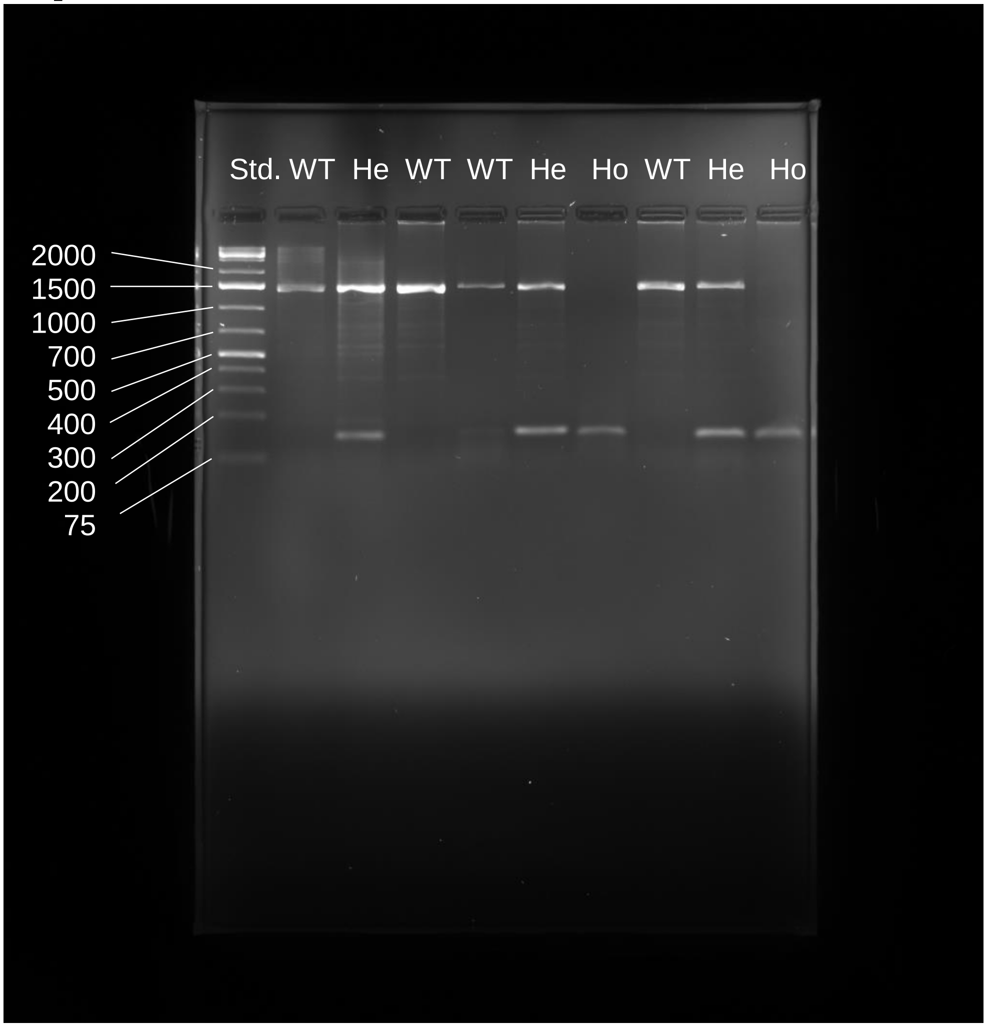

B. *Casp3* wild-type: Sequence confirmation, exon 4 -5 (129/Sv)

GTTCATTTATTCTCAAAGAATAACTATTCTTGTAAAATGCAACTAAGAGCTCTCTCTCTC
TGTCCCAAG**GAATGTCATCTCGCTCTGGTACGGATGTGGACGCAGCCAACCTCAGAGAGA**
CATTCATGGCCTGAAATACCAAGTCAGGAATAAAAATGATCTTACTCGTGAAGACATTT
TGGAATTAATGGATAGTGGTAAGATAGAACCAATTCAAAGCAGGTCCTTCGGAAGCTTCG
GTGGGCGGTGGACAGCAGCCCGCAGCTGGTCAAAGAACAGAGATTAAGTGTCTGCGGAGC
TTAGTCAGAATGGGGCACCTGGATCCCAACCCACACCCAAACCCAAGGCTCAGGCACCAT
CGCAGAAGACAGGCAGAAAGGGTGTAGGAGCAAGGCAGCATCGCTGCTAGCAGGATCCAG
CAGTCCGCATCCTTACACAGAAGGGAGCCCAGGAAGCTATTGACAACTGATGGCTGCTGG
AGGAAGGAGGGCCCACTGTATTTATGGGTGTGGTCCCCGATTGGTTGGCTGTGTTGCAGT
GGATGGGCCCACTCTTGACTGTATATGGCCAGCACAAATTGGACCTTGTGTTATTTTTAT
TTATAAAGGGGAGAGGGGCATAAAGTTGGGTGGAATAGGGGATATATATTTGTGAATATG
CTCAAAATTGCATATATGTAGTTCTCTAGAATGATCTTCATTGGGTAATTTTACTTCATG
GTCTAACTATAGTGATAGAGAGAGTCGAGTATGAAAATTATAGAACTACACTGTACATTA
TTCTGATTGGTTTTTTTGTTTGTTTTTTGTTTTGCTTTGATTTTAAGATACAGTCTCACT
ATTTAGACAGGCTAGCCTCCAGCTTACAAAGATCTGCCTGCCTCTGCTCCCTGTGTGGTG
GGACTAAAGGCATGCGCTACCACCCCCAGCTGCTTATATTTTTTAGTAAAAAGTAAAAAG
AAAGAAAAGAAAATTACGCCATTCCCTAAAAATGGTTCCAAATGTGCTCTGCCTGTGTTA
GACTTGGATGCTCTTGAAATTCAATTTTCAGCATCTTCATCTTGTCATGGTATAACAGTA
ACCCCAGAACTCAGGAAGCTAAGACCATGAAGTCAAAGCTGTCTTCGTCCAGTGAGAAAG
CTAGATACAGATCAGATAAGCCTGGGCTCTTTACTGTATACAGTGCCAGGCTGCCATGTG
CCTTCCCATCTCTGCTCTGTTTTATATTATTTCTGACTGTCTTTCCAG**TTTCTAAGGAAG**
ATCATAGCAAAAGGAGCAGCTTTGTGTGTGTGATTCTAAGCCATGGTGATGAAGGGGTCA
TTTATGGGACAAATGGGCCTGTTGAACTGAAAAAGTTGACTAGCTTCTTCAGAGGCGACT
ACTGCCGGAGTCTGACTGGAAAGCCGAAACTCTTCATCATTCAGGTAGGGCATTGCCCAG
CA**GCTCGGTGCTCAGTTTGGTT**AACTTAGTTAAATGGGCTACAGAACTGCTATATTTTTA

PCR product (WT): 1416 nt

C. *Casp3* deletion PCR product example: 151 nt

GTTCATTTATTCTCAAAGAATAACTATTCTTGTAAAATGCAACTAAGAGCTCTCTCTCTC
TGTCCCAAG**GAATGTCATCTCGCTCTGGTACGGATGTGGACGCAGCCAACCTCAGAG**~~**AGA**~~
~~**CATTCATGGCCTGAAATACCAAGTCAGGAATAAAAATGATCTTACTCGTGAAGACATTT**~~
~~**TGGAATTAATGGATAGTG**GTAAGATAGAACCAATTCAAAGCAGGTCCTTCGGAAGCTTCG~~
~~GTGGGCGGTGGACAGCAGCCCGCAGCTGGTCAAAGAACAGAGATTAAGTGTCTGCGGAGC~~
~~TTAGTCAGAATGGGGCACCTGGATCCCAACCCACACCCAAACCCAAGGCTCAGGCACCAT~~
~~CGCAGAAGACAGGCAGAAAGGGTGTAGGAGCAAGGCAGCATCGCTGCTAGCAGGATCCAG~~
~~CAGTCCGCATCCTTACACAGAAGGGAGCCCAGGAAGCTATTGACAACTGATGGCTGCTGG~~
~~AGGAAGGAGGGCCCACTGTATTTATGGGTGTGGTCCCCGATTGGTTGGCTGTGTTGCAGT~~
~~GGATGGGCCCACTCTTGACTGTATATGGCCAGCACAAATTGGACCTTGTGTTATTTTTAT~~
~~TTATAAAGGGGAGAGGGGCATAAAGTTGGGTGGAATAGGGGATATATATTTGTGAATATG~~
~~CTCAAAATTGCATATATGTAGTTCTCTAGAATGATCTTCATTGGGTAATTTTACTTCATG~~
~~GTCTAACTATAGTGATAGAGAGAGTCGAGTATGAAAATTATAGAACTACACTGTACATTA~~
~~TTCTGATTGGTTTTTTTGTTTGTTTTTTGTTTTGCTTTGATTTTAAGATACAGTCTCACT~~
~~ATTTAGACAGGCTAGCCTCCAGCTTACAAAGATCTGCCTGCCTCTGCTCCCTGTGTGGTG~~
~~GGACTAAAGGCATGCGCTACCACCCCCAGCTGCTTATATTTTTTAGTAAAAAGTAAAAAG~~
~~AAAGAAAAGAAAATTACGCCATTCCCTAAAAATGGTTCCAAATGTGCTCTGCCTGTGTTA~~
~~GACTTGGATGCTCTTGAAATTCAATTTTCAGCATCTTCATCTTGTCATGGTATAACAGTA~~
~~ACCCCAGAACTCAGGAAGCTAAGACCATGAAGTCAAAGCTGTCTTCGTCCAGTGAGAAAG~~
~~CTAGATACAGATCAGATAAGCCTGGGCTCTTTACTGTATACAGTGCCAGGCTGCCATGTG~~
~~CCTTCCCATCTCTGCTCTGTTTTATATTATTTCTGACTGTCTTTCCAG**TTTCTAAGGAAG**~~
~~**ATCATAGCAAAAGGAGCAGCTTTGTGTGTGTGATTCTAAGCCATGGTGATGAAGGGGTCA**~~
~~**TTTATGGGACAAATGGGCCTGTTGAACTGAAAAAGTTGACTAGCTTCTTCAGAGGCGACT**~~
~~**AC**~~**TGCCGGAGTCTGACTGGAAAGCCGAAACTCTTCATCATTCAG**GTAGGGCATTGCCCAG
CA**GCTCGGTGCTCAGTTTGGTT**AACTTAGTTAAATGGGCTACAGAACTGCTATATTTTTA

D.

Caspase-8

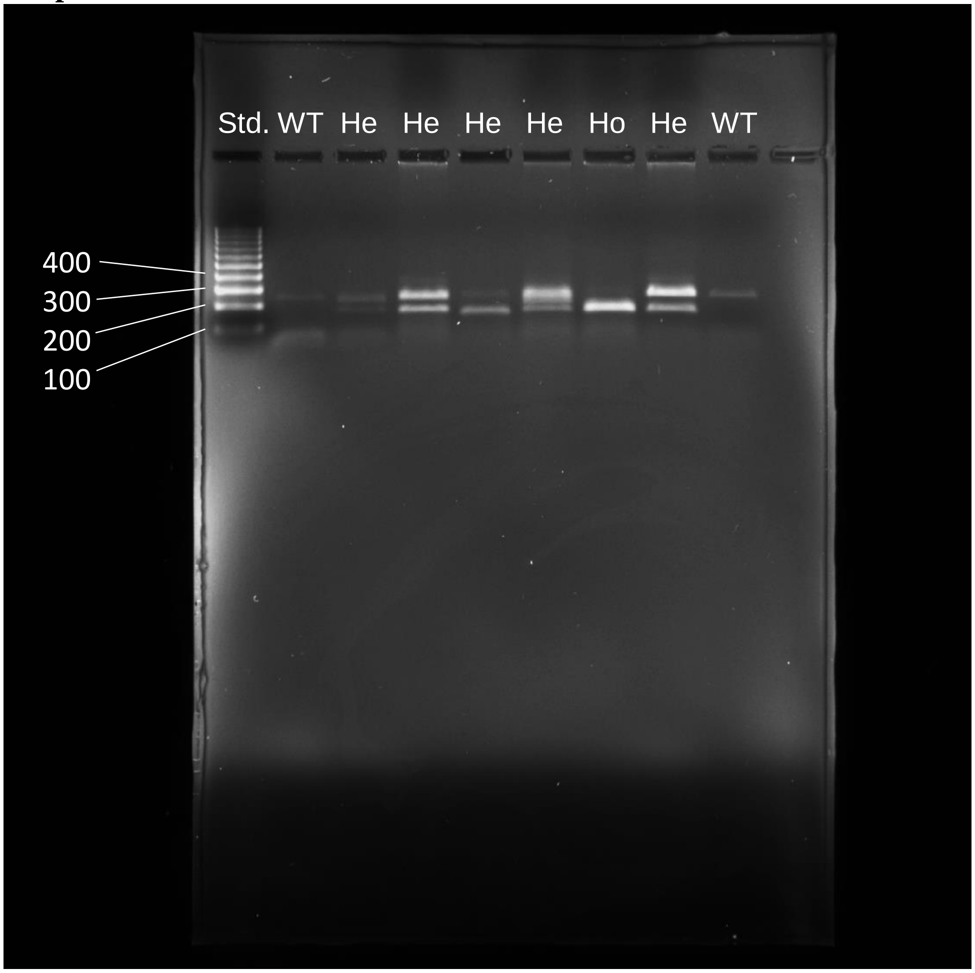

E.

***Casp8* wild-type: Sequence confirmation, exon 3 (129/Sv)**

```
TAACTTCCTCACTTGATCATTAGCATCTTGTGTTGACCCAGGTTACAGCTCTTCTACCTC
TTGATAAGAATGGATTTCCAGAGTTGTCTTTATGCTATTGCTGAAGAACTGGGCAGTGAA
GACCTGGCTGCCCTCAAGTTCCTGTGCTTGGACTACATCCCACACAAGAAGCAGGAGACC
ATCGAGGATGCCCAGAAGCTATTTCTGAGGCTGCGGGAAAAGGGGATGTTGGAGGAAGGC
AATCTGTCTTTCCTGAAAGAGCTGCTTTTCCACATCAGTCGGTGGGACCTGCTGGTCAAC
TTCCTAGACTGCAACCGAGAGGAGATGGTGAGAGAGCTGCGGGATCCAGACAATGCCCAG
ATTTCTCCCTACAGGTGGGTAGAAAGGCTGTGGTGGGGGGACTGGGAAGTGTGGGCTGAA
```

PCR product (WT): 261 nt

F.

***Casp8* deletion PCR product example: 169 nt**

TAACTTCCTCACTTGATCATTAGCATCTTGTGTTGACCCAGGTTACAGCTCTTCTACCTC
TTGATAAGAATGGATTTCCAGAGTTGTCTTTATGCTATTGCTGAAGAACTGGGCAGTGAA
GACCTGGCTGCCCTCAAGTTCCTGTGCTTGGACTACATCCCACACAAGAAGCAGGAGACC
~~ATCGAGGATGCCCAGAAGCTATTTCTGAGGCTGCGGGAAAAGGGGATGTTGGAGGAAGGC~~
~~AATCTGTCTTTCCTGAAAGAGCTGCTTTTCCACAT~~CAGTCGGTGGGACCTGCTGGTCAAC
TTCCTAGACTGCAACCGAGAGGAGATGGTGAGAGAGCTGCGGGATCCAGACAATGCCCAG
ATTTCTCCCTACAGGTGGGTAGAAAGGCTGTGGTGGGGGGACTGGGAAGTGTGGGCTGAA

G.

MLKL

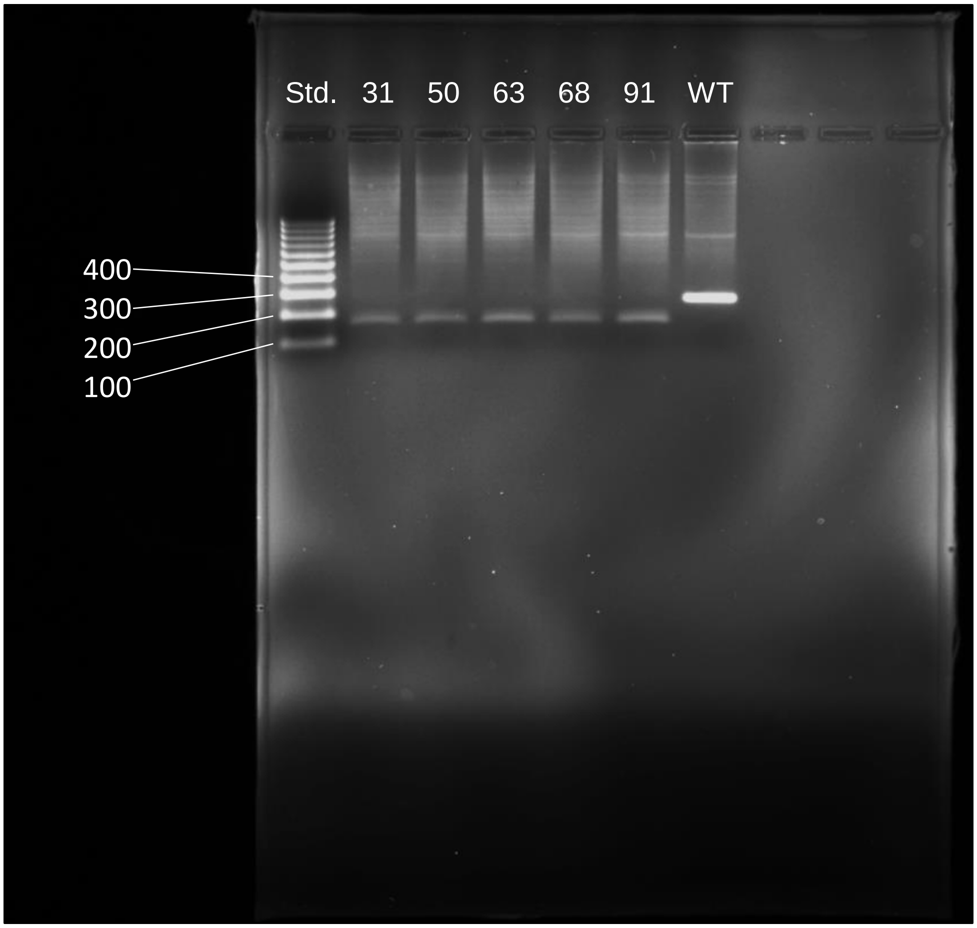

H. *MLKL* wild-type: Sequence confirmation, exon 2 (129/Sv)

CAGGGGATTGTGGTATTTCAACCAGTCTATGCATCTCTTTCAGCTATGGATAAATTGGGA
CAGATCATCAAGTTAGGCCAGCTCATCTATGAACAGTGTGAAAAGATGAAATACTGCCGG
AAACAATGCCAGCGTCTAGGAAACCGTGTGCACGGCCTGCTACAGCCTCTCCAGAGACTC
CAGGCCCAAGGAAAGAAGAACCTGCCCGATGACATTACTGCTGCCCTGGGCCGTTTTGAT
GAAGTCCTGAAGGAGGCTAACCAGCAGATAGAAAAGTTCAGCAAGAAGTCCCATATTTGG
AAGTTTGTGAGTGTGGGCAATGATAAGATCCTCTTCCATGAAGTGAATGAGAAGCTGAGA
GACGTCTGGGAGGAGCTGTTGCTGCTGCTTCAGGTTTATCATTGGAATACCGTTTCAGAT
GTCAGCCAGCCAGCATCCTGGCAGCAGGAAGATCGACAGGATGCAGAGGAAGACGGGTGA

PCR product (WT): 270 nt

I. *MLKL* deletion PCR product example: 171 nt

CAGGGGATTGTGGTATTTCAACCAGTCTATGCATCTCTTTCAGCTATGGATAAATTGGGA
CAGATCATCAAGTTAGGCCAGCTCATCTATGAACAGTGTGAAAAGATGAAATACTGCCGG
AAACAATGCCAGCG~~TCTAGGAAACCGTGTGCACGGCCTGCTACAGCCTCTCCAGAGACTC~~
~~CAGGCCCAAGGAAAGAAGAACCTGCCCGATGACATTACTGCTGCCCTGG~~GCCGTTTTGAT
GAAGTCCTGAAGGAGGCTAACCAGCAGATAGAAAAGTTCAGCAAGAAGTCCCATATTTGG
AAGTTTGTGAGTGTGGGCAATGATAAGATCCTCTTCCATGAAGTGAATGAGAAGCTGAGA
GACGTCTGGGAGGAGCTGTTGCTGCTGCTTCAGGTTTATCATTGGAATACCGTTTCAGAT
GTCAGCCAGCCAGCATCCTGGCAGCAGGAAGATCGACAGGATGCAGAGGAAGACGGGTGA

Supplemental Figure S3. Clones and sequence analysis of ES cell lines generated ME via transfection for CRISPR-mediated gene targeting for several genetic loci.
(**A**) Representative example of allelic frequency, CRISPR- mediated targeting of caspase-3 gene locus. (**B**) Verification of wild-type sequence of identified caspase-3 gene segment (exon 4-5) with expected PCR product shown. For each sequence, wild-type / mutant sequencing primers are indicated in blue, CRISPRs utilized are indicated in yellow and associated PAM sequence in purple. Gene exons are indicated in tan. (**C**) Typical deletion obtained using CRISPRs indicated in *Casp3* locus with observed PCR product shown. Deleted nucleotides are indicated via strikethrough. Nucleotides falling outside of traditional CRISPR cleavage site (between nucleotides 3-4 beyond PAM site) are indicated in green. (**D**) Representative example of allelic frequency, CRISPR- mediated targeting of caspase-8 gene locus. (**E**) Verification of wild-type sequence of identified caspase-8 exon 3 with expected PCR product shown. Translation start site ATG indicated in underline, sequencing primers are indicated in blue, CRISPRs utilized are indicated in yellow with associated PAM sequence in purple. Gene exon is indicated in tan. (**F**) Typical deletion obtained using CRISPRs indicated for *Casp8* locus with observed PCR product shown. Deleted nucleotides are indicated via strikethrough. Nucleotides falling outside of traditional CRISPR site (nucleotides 3-4 beyond PAM site) are indicated in green. (**G**) Example of bi-allelic homozygous mutant (null) lines for the MLKL locus via CRISPR- mediated ME targeting. (**H**) Verification of wild-type sequence of identified caspase-8 exon 2 with expected PCR product shown. Translation start site ATG indicated in underline, sequencing primers are indicated in blue, CRISPRs utilized are indicated in yellow with associated PAM sequence in purple. Gene exon is indicated in tan. (**I**) Typical deletion obtained using CRISPRs indicated for *MLKL* locus with observed PCR product shown. Deleted nucleotides are indicated via strikethrough. Nucleotides falling outside of traditional CRISPR site (nucleotides 3-4 beyond PAM site) are indicated in green.

Supplemental Figure S4.

A.

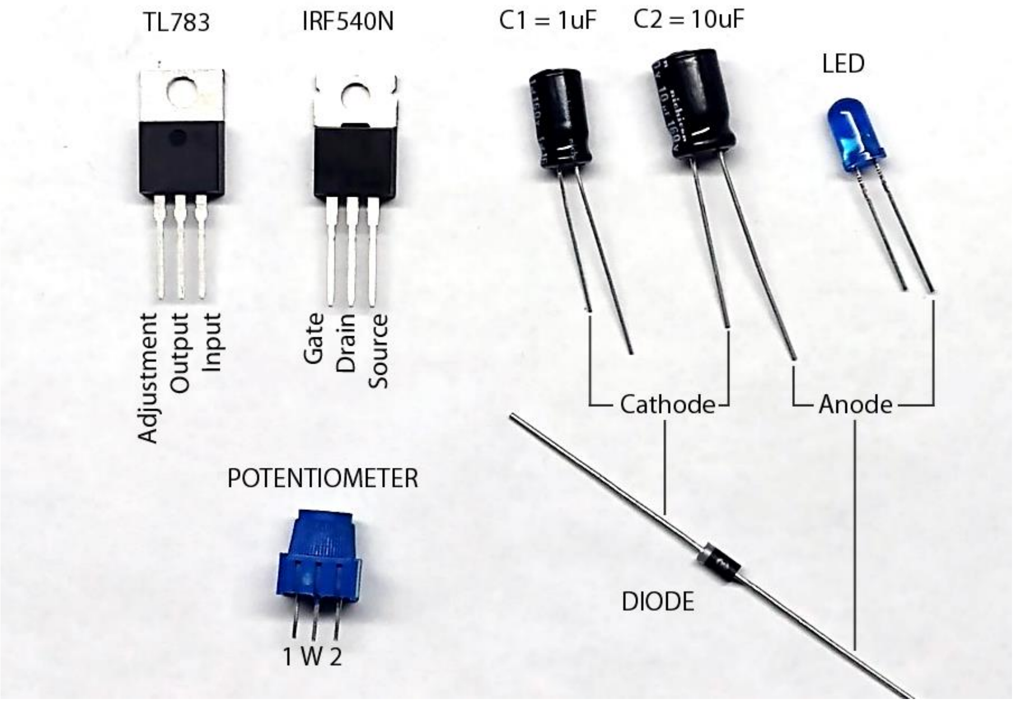

B.

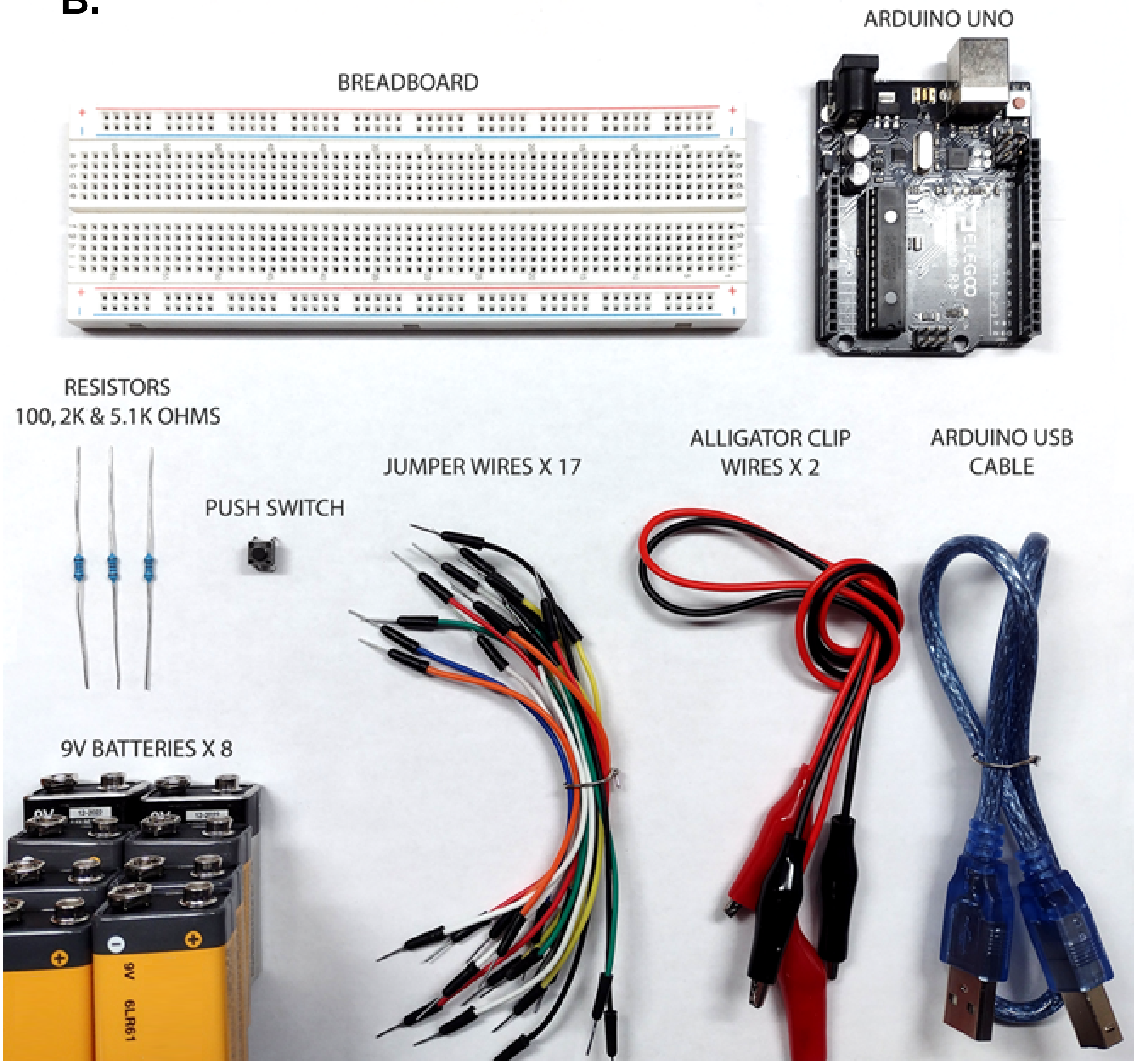

Supplemental Figure S4. Electroporator materials. (**A**) Polarized electrical components or those with specific pin functions are shown and labelled. TL783 and IRF540N pins are labelled as shown with the casing facing upwards. Capacitors (C1, C2) and LEDs are typically constructed with the longer leg being the anode and shorter being the cathode. The cathode of the diode can be identified from the grey stripe on body of the diode. The potentiometer pins are labelled with the notched surface facing upwards. (**B**) Additional / non-polarized components.

Supplemental Figure S5.

A.

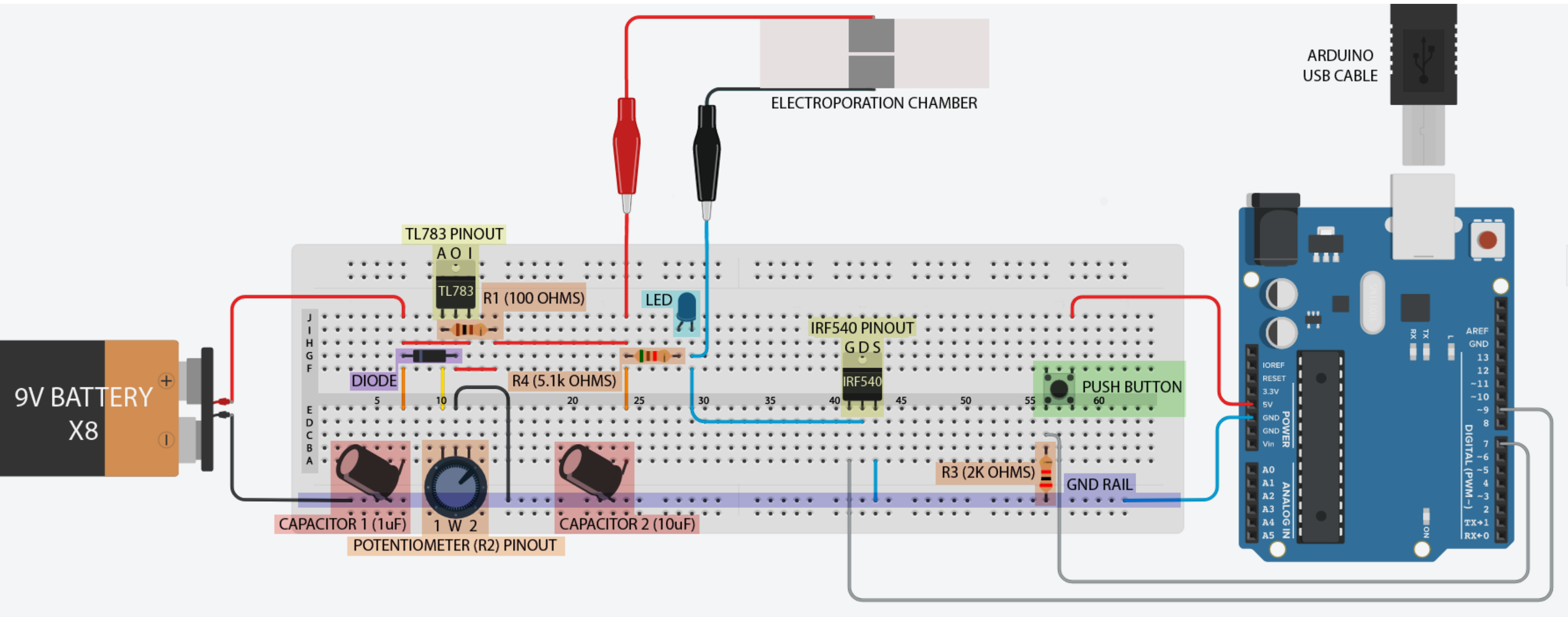

B.

Momentary_Switch_for_Electroporation | Arduino 1.8.13 (Windows Store 1.8.42.0)

File Edit Sketch Tools Help — Click this button to upload the code to the Arduino

Momentary_Switch_for_Electroporation §

```
//Momentary Switch for Electroporation. Arduino IDE ver. 1.8.13
//Author: Shudi Huang
//Date: Aug 9, 2021

byte switchInput=7;
byte MOSFEToutput=9;
int pulseDura=1;//<------------------This is the duration (in milliseconds by default) of the electric pulse. Change this value to change the duration.

void setup() {
  pinMode(switchInput,INPUT);
  pinMode(MOSFEToutput,OUTPUT);
}

void loop() {
  if(digitalRead(switchInput)==HIGH){//Turns MOSFET on/off after certain delay
    digitalWrite(MOSFEToutput,HIGH);
    delay(pulseDura);//<--------------To achieve microsecond length pulses, change "delay(pulseDura)" to "delayMicroseconds(pulseDura)"
    digitalWrite(MOSFEToutput,LOW);

    do{
      delay(25);
    }while(digitalRead(switchInput)==HIGH);//Prevents triggering MOSFET again until switch is released
  }
}
```

C.

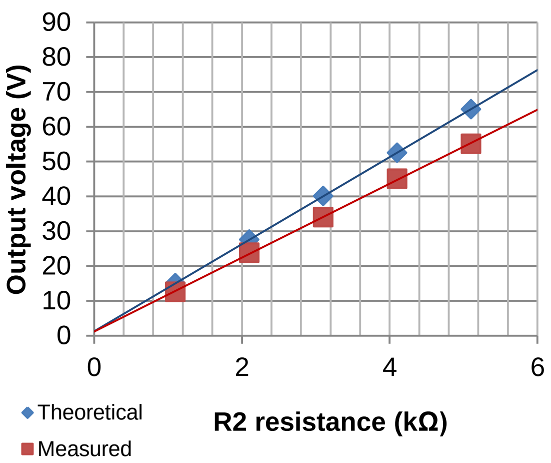

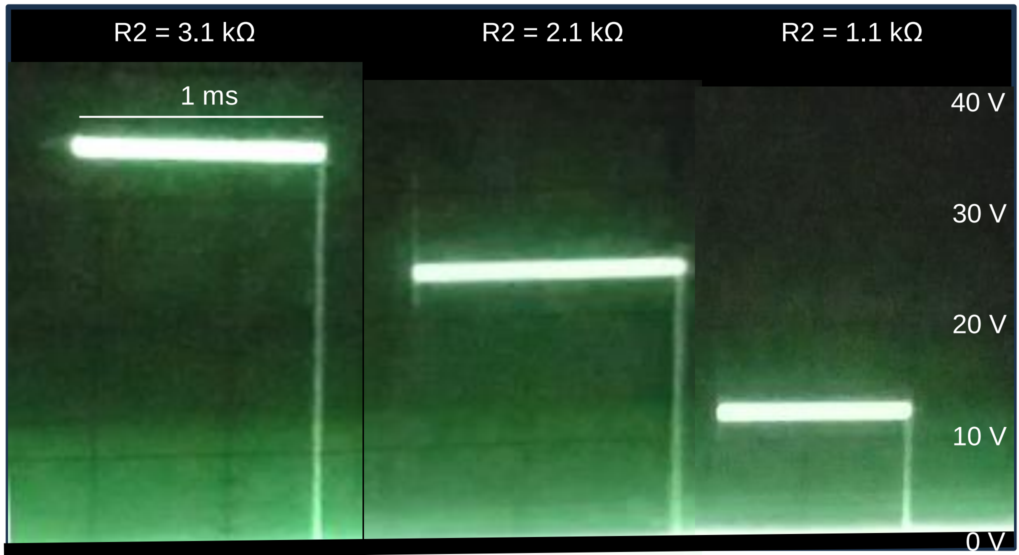

Supplemental Figure S5. Electroporator construction details. (**A**) Layout of ME circuit board and Arduino, with electroporator chamber and power supply connections indicated, corresponding to Figure 5B and provided breadboard pin placement instructions. Orientation of relative breadboard positions indicated by row letter and column number. (**B**) Explicit code for 'momentary switch' program for single pulse electroporation. Code shown programs Arduino to read inputs from the push button (digital pin 7), outputting a signal to the IRF540N MOSFET (digital pin 9). Instructions to change signal duration are indicated in blue. (**C**) Theoretical versus experimentally measured square-wave output for different fixed resistance values of R2 with oscilloscope trace examples.
