# Supplementary material for: An efficient low cost means of biophysical gene transfection in primary cells
Source: Sci Rep. 2024 Jun 8;14:13179. doi: 10.1038/s41598-024-62996-y (PMC11161637; doi:10.1038/s41598-024-62996-y)
Supplement: Supplementary file 2 — Supplementary Information 2. [file 41598_2024_62996_MOESM2_ESM.docx]

Below are the contents of the file “Momentary_Switch_for_Electroporation.ino” in its entirety.

//Momentary Switch for Electroporation. Arduino IDE ver. 1.8.13

//Author: Shudi Huang

//Date: Aug 9, 2021

byte switchInput=7;

byte MOSFEToutput=9;

int pulseDura=1;//<-------------------This is the duration (in milliseconds by default) of the electric pulse. Change this value to change the duration.

void setup() {

pinMode(switchInput,INPUT);

pinMode(MOSFEToutput,OUTPUT);

}

void loop() {

if(digitalRead(switchInput)==HIGH){//Turns MOSFET on/off after certain delay

digitalWrite(MOSFEToutput,HIGH);

delay(pulseDura);//<--------------To achieve microsecond length pulses, change "delay(pulseDura)" to "delayMicroseconds(pulseDura)"

digitalWrite(MOSFEToutput,LOW);

do{

delay(25);

}while(digitalRead(switchInput)==HIGH);//Prevents triggering MOSFET again until switch is released

}

}
